# HIV and cancer: a comparative retrospective study of Brazilian and U.S. clinical cohorts

**DOI:** 10.1186/1750-9378-10-4

**Published:** 2015-02-02

**Authors:** Jessica L Castilho, Paula M Luz, Bryan E Shepherd, Megan Turner, Sayonara R Ribeiro, Sally S Bebawy, Juliana S Netto, Catherine C McGowan, Valdiléa G Veloso, Eric A Engels, Timothy R Sterling, Beatriz Grinsztejn

**Affiliations:** Department of Medicine, Division of Infectious Diseases, MCN A2200, Vanderbilt University School of Medicine, 1611 21st Avenue South, Nashville, TN 37232 USA; Fundação Oswaldo Cruz, Instituto Nacional de Infectologia Evandro Chagas, HIV/AIDS Clinical Research Center, Avenida Brasil 4365, Rio de Janeiro, RJ CEP: 21040-360 Brazil; Vanderbilt University School of Medicine Department of Biostatistics, 2525 West End, Suite 11000, Nashville, TN 37203 USA; Division of Cancer Epidemiology and Genetics, National Cancer Institute, 9609 Medical Center Drive, MSC 9776, Bethesda, MD 20892 USA

**Keywords:** HIV, Malignancy, Cancer, Brazil, Anal cancer, Kaposi sarcoma, Non-Hodgkin lymphoma, Lung cancer, Age, Sex

## Abstract

**Background:**

With successful antiretroviral therapy, non-communicable diseases, including malignancies, are increasingly contributing to morbidity and mortality among HIV-infected persons. The epidemiology of AIDS-defining cancers (ADCs) and non-AIDS-defining cancers (NADCs) in HIV-infected populations in Brazil has not been well described. It is not known if cancer trends in HIV-infected populations in Brazil are similar to those of other countries where antiretroviral therapy is also widely available.

**Methods:**

We performed a retrospective analysis of clinical cohorts at Instituto Nacional de Infectologia Evandro Chagas (INI) in Rio de Janeiro and Vanderbilt Comprehensive Care Clinic (VCCC) in Nashville from 1998 to 2010. We used Poisson regression and standardized incidence ratios (SIRs) to examine incidence trends. Clinical and demographic predictors of ADCs and NADCs were examined using Cox proportional hazards models.

**Results:**

This study included 2,925 patients at INI and 3,927 patients at VCCC. There were 57 ADCs at INI (65% Kaposi sarcoma), 47 at VCCC (40% Kaposi sarcoma), 45 NADCs at INI, and 82 at VCCC. From 1998 to 2004, incidence of ADCs remained statistically unchanged at both sites. From 2005 to 2010, ADC incidence decreased in both cohorts (INI incidence rate ratio per year = 0.74, *p* < 0.01; VCCC = 0.75, *p* < 0.01). Overall Kaposi sarcoma incidence was greater at INI than VCCC (3.0 vs. 1.2 cases per 1,000 person-years, *p* < 0.01). Incidence of NADCs remained constant throughout the study period (overall INI incidence 3.6 per 1,000 person-years and VCCC incidence 5.3 per 1,000 person-years). Compared to general populations, overall risk of NADCs was increased at both sites (INI SIR = 1.4 [95% CI 1.1-1.9] and VCCC SIR = 1.3 [1.0-1.7]). After non-melanoma skin cancers, the most frequent NADCs were anal cancer at INI (n = 7) and lung cancer at VCCC (n = 11). In multivariate models, risk of ADC was associated with male sex and immunosuppression. Risk of NADC was associated with increased age.

**Conclusions:**

In both cohorts, ADCs have decreased over time, though incidence of KS was higher at INI than VCCC. Rates of NADCs remained constant over time at both sites.

**Electronic supplementary material:**

The online version of this article (doi:10.1186/1750-9378-10-4) contains supplementary material, which is available to authorized users.

## Background

In the era of highly active antiretroviral therapy (ART), cancer is an important cause of morbidity and mortality of individuals living with HIV. Since 1996, in high-income countries, non-AIDS-defining cancers (NADCs) have increasingly replaced AIDS-defining cancers (ADCs) as causes of cancer morbidity in HIV-infected populations [1]. In particular, Hodgkin lymphoma, liver cancer, lung cancer, anal cancer, and non-melanoma skin cancers occur at rates in HIV-infected populations above expected in general populations [2–8].

Cancers occurring in HIV-infected populations in low- and middle-income countries have not been as well described. Despite increasing availability of ART, ADCs continue to account for substantial morbidity and mortality [9, 10]. In a recent study from Latin America, ADCs were the most common cancers diagnosed in patients following initiation of ART [11]. However, even in resource-limited settings, NADCs and other non-communicable diseases are of growing concern. There is a recognized global need for improved screening, diagnosis, and treatment programs for cancer in HIV-infected populations [12–14].

Brazil represents an important upper-middle-income country to evaluate the epidemiology of cancer occurring in HIV-infected populations. Similar to high-income countries, ART has been available in Brazil since 1996. Brazil also has cancer registries to compare rates of specific cancer types in HIV-infected populations to rates in the general population. Importantly, recent studies from Brazil have indicated that non-AIDS-defining conditions increasingly contribute to morbidity and mortality in HIV-infected individuals [15–19]. The epidemiology of ADCs and NADCs in HIV-infected populations has not been described in Brazil. It is unknown whether cancer trends in HIV-infected populations in Brazil are similar to those in high-income countries like the U.S. This study aimed to compare the epidemiology and predictors of malignancy in clinical cohorts of HIV patients from Rio de Janeiro (Instituto Nacional de Infectologia Evandro Chagas [INI]), Brazil, and Tennessee (Vanderbilt Comprehensive Care Clinic [VCCC]), USA, as an example referent HIV-infected population with a diverse patient profile.

## Results

There were 2,925 patients (12,333.8 person-years of follow-up) at INI and 3,927 patients (15,327.2 person-years of follow-up) at VCCC who met inclusion criteria for the study period. Demographic and clinical characteristics of the cohorts are described in Table 1. Overall, patients at INI were more likely to be female, slightly younger, and have lower CD4+ lymphocyte count at clinic entry. INI patients were more likely to report heterosexual risk for HIV and to enroll in care in later calendar years. VCCC patients were more likely to report injection drug use, have hepatitis C virus infection, and report antiretroviral therapy (ART) exposure prior to clinic enrollment. Of those patients surveyed, slightly over half reported a history of tobacco use at each site. Patients at both sites were followed on average for approximately three years. A high proportion of patients at both clinics received ART during follow-up. While more patients at INI had missing laboratory values at clinic enrollment, the frequency of laboratory monitoring during follow-up was similar between the two sites (data not shown).

**Table 1:** **Demographic and clinical characteristics of the study participants by site**

	INI	VCCC	***P***value ^a^
	N = 2,925	N = 3,927	
Female sex (%)	978 (33)	938 (24)	<0.01
HIV transmission risk categories^b^ (%)			<0.01
MSM	1,035 (35)	1,569 (40)	
IDU	41 (1)	374 (10)	
Heterosexual males	714 (24)	456 (12)	
Heterosexual females	969 (33)	835 (21)	
All others	166 (6)	693 (18)	
Median age in years at clinic entry [IQR]	36 [29–43]	38 [31–45]	<0.01
Race (%)			0.69
White	1,552 (53)	2,103 (54)	
Non-white	1,373 (47)	1,824 (46)	
Median CD4+ lymphocyte count at clinic entry (cells/μL) [IQR]	287 [106–504]	325 [153–518]	<0.01
Missing CD4+ lymphocyte count at clinic entry (%)	884 (30)	346 (9)	<0.01
Median log_10_ HIV-1 RNA level at clinic entry (copies/mL) [IQR]	4.5 [3.6-5.2]	4.3 [3.1-5.0]	<0.01
Missing HIV-1 RNA at clinic entry (%)	1,364 (47)	322 (8)	<0.01
Median year of clinic entry [IQR]	2006 [2003–2008]	2004 [2001–2007]	<0.01
Hepatitis C virus infection^c^ (%)	243 (9)	537 (14)	<0.01
Missing hepatitis C virus infection data (%)	216 (7)	0	<0.01
Hepatitis B virus infection^c^ (%)	160 (6)	216 (6)	0.13
Missing hepatitis B virus infection data (%)	433 (15)	0	<0.01
History of tobacco use^d^ (%)	1,131/2,129 (53)	1,319/2,155 (61)	<0.01
Missing tobacco history (%)	796 (27)	1,772 (45)	<0.01
History of cancer prior to clinic entry (%)	56 (2)	105 (3)	0.04
History of ART exposure prior to clinic entry (%)	759 (26)	1,566 (40)	<0.01
Median follow-up time in years [IQR]	3.5 [1.5-6.1]	3.1 (1.1-6.5)	<0.01
Receipt of any ART during follow-up (%)	2,158 (74)	3,145 (80)	<0.01

^a^ *P* value result refers to results of Chi square test (categorical and binary variables) or Wilcoxon rank sum test (continuous variables).

^b^HIV transmission risk categories are mutually exclusive.

^c^Hepatitis C and hepatitis B infection status at clinic entry for VCCC and at any point obtained during follow-up at INI, as many patients there did not have serologic testing until after clinic entry.

^d^Tobacco use was available from single, cross-sectional surveys completed by some patients at both clinic sites that was performed without relation to this study.

Abbreviations:

INI: Instituto Nacional de Infectologia Evandro Chagas, Fundação Oswaldo Cruz, Rio de Janeiro, RJ, Brazil.

VCCC: Vanderbilt Comprehensive Care Clinic, Nashville, TN, USA.

MSM: men who have sex with men.

IDU: injection drug use.

ART: antiretroviral therapy.

During the study period, there were 57 incident ADCs (37 cases of Kaposi sarcoma [KS] and 20 cases of non-Hodgkin lymphoma [NHL]) and 45 incident NADCs diagnosed at INI. There were 47 incident ADCs (19 KS, 27 NHL, and 1 cervical cancer) and 82 incident NADCs diagnosed at VCCC. The incidence of ADCs during the study period was 4.6 per 1,000 person-years (95% confidence interval [CI] 3.5-6.0) at INI and 3.3 per 1,000 person-years (95% CI 2.5-4.4) at VCCC. The incidence of NADCs (including non-melanoma skin cancers) during the study period was 3.6 per 1,000 person-years (95% CI 2.7-4.9) at INI and 5.3 per 1,000 person-years (95% CI 4.3-6.4) at VCCC. The incidence of ADCs and NADCs by year and site are shown in Additional file 1: Table S1 and Additional file 2: Table S2, respectively. At INI, from 1998 to 2010, incidence of ADCs decreased from 38.3 to 1.4 per 1,000 person-years (incidence rate ratio [IRR] per year = 0.92 [95% CI 0.84-1.00]), which was driven by declining incidence of KS (KS IRR per year = 0.89 [95% CI 0.83-0.97]; NHL IRR per year = 0.96 [95% CI 0.82-1.11]). When results were restricted to 2005 to 2010, the trend of decreasing incidence was even stronger (ADC IRR per year = 0.74 [95% CI 0.65-0.85]). Similarly, rates of ADCs at VCCC decreased from 5.7 to 1.3 per 1,000 person-years from 1999 to 2010 (IRR per year = 0.90 [95% CI 0.85-0.96]), also due to changing rates of KS (KS IRR per year 0.84 [95% CI 0.77-0.92]; NHL IRR per year 0.94 [95% CI 0.83-1.06]). Like INI, this trend was strongest in the years 2005 to 2010 (ADC IRR per year = 0.75 [95% CI 0.67-0.84]). At both sites, incidence rates of ADCs were statistically unchanged from 1998 to 2004 (INI IRR per year = 0.82 [95% CI 0.61-1.12] and VCCC IRR per year = 0.91 [95% CI 0.80-1.04]). Incidence of NADCs remained constant at both sites overtime (INI IRR per year = 0.99 [95% CI 0.89-1.12]; VCCC IRR per year = 1.00 [95% CI 0.93-1.07]). The overall incidence of KS was over two-fold higher at INI than at VCCC during the study period (3.0 vs. 1.24 per 1,000 person-years, *p* < 0.01).

Rates of a number of cancers were higher than expected in respective general populations, with overall ADC and NADC standardized incidence ratios [SIRs] greater than one at both sites (Table 2). For both sites, rates of KS, NHL, and anal cancer were significantly increased compared to respective general populations. Cervical cancer rates at both sites were not increased. At INI, SIRs for lung cancer, squamous and basal cell skin cancers, and laryngeal cancer were increased and nearly met statistical significance. At VCCC, lung cancer and biliary tract cancers occurred at rates significantly above expected. Liver cancer was also associated with increased risk but did not meet statistical significance at VCCC. There was no case of liver cancer in the INI cohort.

**Table 2:** **Standardized incidence ratios (SIRs) of AIDS- and non-AIDS-defining cancers by study site**

	INI		VCCC	
	N	SIR [95% CI]	N	SIR [95% CI]
AIDS-defining cancers	57	24.6 [18.7-31.9]	47	17.0 [12.5-22.6]
Kaposi Sarcoma	37	269 [189–371]	19	1157 [697–1807]
Non-Hodgkin Lymphoma	20	21.2 [12.9-32.7]	27	11.4 [7.5-16.6]
Cervix	0	0 [0–3.2]	1	2.7 [0.7-14.9]
Non-AIDS-defining cancers	45	1.4 [1.1-1.9]	81	1.3 [1.0-1.7]
Oral Cavity & Pharynx	3	1.7 [0.4-5.0]	2	1.2 [0.1-4.3]
Esophagus	1	1.0 [0–5.4]	1	2.1 [0.1-11.9]
Stomach	2	1.5 [0.2-5.5]	0	0 [0–5.2]
Small Intestine	0	0 [0–47.9]	1	4.2 [0.1-23.6]
Colon & Rectum	2	1.1 [0.1-4.1]	5	1.2 [0.4-2.9]
Anus	7	62.3 [25.1-128]	9	25.0 [11.4-47.5]
Liver	0	0 [0–8.6]	3	3.7 [0.8-10.7]
Biliary Tract	0	0 [0–19.4]	2	17.6 [2.1-63.5]
Pancreas	1	2.9 [0.1-16.1]	0	0 [0–4.8]
Larynx	3	4.6 [0.9-13.3]	2	4.4 [0.5-15.7]
Lung & Bronchus	4	3.0 [0.8-7.7]	11	2.8 [1.4-5.1]
Mediastinum	1	23.0 [0.6-128]	0	0 [0–161]
Soft Tissue Sarcoma	0	0 [0–1.06]	1	2.4 [0.1-13.4]
Squamous and basal cell skin cancers	9	1.9 [0.9-3.7]	28	Not available
Melanoma	0	0 [0–8.1]	2	0.6 [0.1-2.1]
Breast	5	1.3 [0.4-2.9]	1	0.2 [0–1.3]
Corpus Uterus	1	5.5 [0.1-30.8]	0	0 [0–7.7]
Ovary	1	3.0 [0.1-16.5]	1	3.1 [0.1-17.2]
Prostate	1	0.3 [0–1.6]	4	0.4 [0.1-1.0]
Testis	0	0 [0–16.1]	0	0 [0–4.7]
Urinary Bladder	0	0 [0–8.3]	3	2.6 [0.5-7.5]
Kidney	2	4.5 [0.5-16.2]	0	0 [0–2.2]
Brain & other CNS	0	0 [0–4.3]	0	0 [0–4.8]
Thyroid	0	0 [0–8.8]	0	0 [0–2.5]
Other Endocrine	0	0 [0–115]	1	10.0 [0.3-55.5]
Hodgkin Lymphoma	0	0 [0–12.2]	1	1.9 [0.1-10.5]
Myeloma	0	0 [0–12.3]	1	1.6 [0–9.0]
Leukemia	1	1.9 [0.1-10.7]	1	1.0 [0–5.6]
Mesothelioma	0	0 [0–181]	0	0 [0–84.9]
Other	1	1.0 [0–5.3]	1	1.7 [0–9.6]

Comparator population for INI SIRs: Belo Horizonte, MG, general population.

Comparator population for VCCC SIRs: Atlanta, GA, general population.

Abbreviations:

INI: Instituto Nacional de Infectologia Evandro Chagas, Oswaldo Cruz, Rio de Janeiro, RJ, Brazil.

VCCC: Vanderbilt Comprehensive Care Clinic, Nashville, TN, USA.

CI: Confidence interval.

Cox proportional hazards models for predictors of first ADC, stratified by cohort, are shown in Table 3. In univariable analyses, male sex, increased age at clinic entry, low or missing CD4+ lymphocyte nadir, low time-varying CD4+ lymphocyte count, increased log_10_ HIV RNA, and cumulative follow-up time with log_10_ HIV RNA ≥ 5.0 were all associated with increased risk of ADC among patients at INI. Similar predictors were observed at VCCC with the exception that age was not associated with risk while race was. In multivariable models, male sex and low CD4+ lymphocyte count remained statistically associated with increased risk of ADC at both sites. At INI, increased age at clinic entry also remained statistically significant. Cumulative follow-up time with log_10_ HIV RNA ≥ 5.0 was no longer associated with ADC risk when included in a model adjusting for CD4+ lymphocyte count.

**Table 3:** **Univariable and multivariable Cox proportional hazards models for predictors of first AIDS-defining cancer diagnosis**

	INI (n = 57)							VCCC (n = 47)						
	N ^#^	HR	95% CI	***P***value	aHR	95% CI	***P***value	N ^#^	HR	95% CI	***P***value	aHR	95% CI	***P***value
Female sex (ref = male)	57	0.3	0.1-0.6	<0.01	0.3	0.1-0.7	<0.01	47	0.3	0.1-0.8	0.02	0.3	0.1-0.8	0.02
HIV transmission risk categories														
MSM (ref)	28	1.0						24	1.0					
IDU	2	1.6	0.4-6.7	0.51				6	1.1	0.4-2.6	0.90			
Heterosexual males	14	0.7	0.4-1.4	0.31				3	0.4	0.1-1.4	0.17			
Heterosexual females	76	0.3	0.1-0.6	<0.01				4	0.3	0.1-0.9	0.03			
All others	6	1.6	0.7-3.9	0.29				10	1.2	0.6-2.5	0.64			
Age at clinic entry (per 10 years)	57	1.3	1.1-1.6	0.01	1.3	1.0-1.3	0.04	47	0.9	0.7-1.1	0.30	0.8	0.6-1.1	0.16
Non-white race (ref = white)	57	1.1	0.7-1.9	0.64				47	0.5	0.3-0.9	0.03			
CD4+ lymphocyte count nadir (cells/μL)^§^														
≥200 (ref)	10	1.0						14	1.0					
<200	39	4.6	2.3-9.4	<0.01				31	3.7	1.9-7.2	<0.01			
Missing	8	3.3	1.3-7.9	0.01				2	1.3	0.3-5.8	0.73			
CD4+ lymphocyte count (cells/μL)^§^														
≥200 (ref)	12	1.0			1.0			13	1.0			1.0		
50-199	16	6.8	3.2-14.7	<0.01	5.8	2.6-13.0	<0.01	8	3.0	1.2-7.3	0.02	2.9	1.1-7.5	0.03
<50	14	16.5	7.2-37.6	<0.01	13.7	5.9-31.9	<0.01	13	10.6	4.9-23.2	<0.01	9.5	4.0-22.8	<0.01
Missing	15	3.8	1.8-8.0	<0.01	3.9	1.8-8.5	<0.01	13	1.3	0.6-2.9	0.52	1.4	0.6-3.4	0.39
Log_10_ HIV RNA level (per unit)^§^	37	1.2	1.1-1.3	<0.01				34	1.7	1.2-2.3	<0.01			
Cumulative time of log_10_ HIV RNA ≥ 5.0 (per 6 months)^§^	57	1.5	1.1-1.9	<0.01	1.2	0.9-1.6	0.31	47	1.4	1.2-1.7	<0.01	1.1	0.8-1.4	0.55
Year of clinic enrollment	57	1.0	0.9-1.1	0.98	1.0	0.9-1.1	0.67	47	1.0	0.9-1.0	0.19	1.0	0.9-1.1	0.56
Hepatitis C virus infection	52	1.2	0.5-2.8	0.70				47	0.8	0.4-2.0	0.70			
Hepatitis B virus infection	50	1.7	0.7-4.2	0.28				47	1.0	0.3-3.2	0.96			
History of tobacco use	28	1.0	0.5-2.1	1.00				25	1.5	0.6-3.5	0.38			
Cancer prior to clinic entry	57	0	-	-				47	2.6	0.8-8.4	0.11			
ART exposure prior to clinic entry	57	1.4	0.8-2.4	0.23				47	0.7	0.4-1.3	0.25			

^§^Time-updated covariate.

^#^Number of events included in model.

Abbreviations:

INI: Instituto Nacional de Infectologia Evandro Chagas, Fundação Oswaldo Cruz, Rio de Janeiro, RJ, Brazil.

VCCC: Vanderbilt Comprehensive Care Clinic, Nashville, TN, USA.

HR: hazard ratio.

aHR: adjusted hazard ratio.

CI: confidence interval.

MSM: men who have sex with men.

IDU: injection drug use.

ART: antiretroviral therapy.

The Cox models for predictors of first NADC, excluding squamous and basal cell skin cancers, are shown in Table 4. In univariable analyses, at INI, only other HIV transmission risk category and increased age at clinic entry were statistically associated with risk of NADCs. Time-varying CD4+ lymphocyte count was not associated with NADC risk. At VCCC, heterosexual females, compared with men who have sex with men, had a decreased risk of NADCs. Age, low CD4+ lymphocyte nadir, and history of malignancy prior to clinic entry were associated with increased risk of NADC at VCCC in univariable analyses. In multivariable, only increasing age at clinic entry remained statistically associated with risk of incident NADC in either cohort.

**Table 4:** **Univariable and multivariable Cox proportional hazards models for predictors of first non-AIDS-defining cancer diagnosis, excluding squamous and basal cell skin cancers**

	INI (n = 36)							VCCC (n = 50)						
	N ^#^	HR	95% CI	***P***value	aHR	95% CI	***P***value	N ^#^	HR	95% CI	***P***value	aHR	95% CI	***P***value
Female sex (ref = male)	36	1.3	0.6-2.5	0.46	1.4	0.7-2.7	0.31	50	0.6	0.3-1.2	0.15	0.7	0.3-1.6	0.38
HIV transmission risk categories														
MSM (ref)	6	1.00						28	1.0					
IDU	1	3.3	0.4-26.2	0.26				4	0.6	0.2-1.7	0.33			
Heterosexual males	10	2.4	0.9-6.6	0.09				8	1.0	0.4-2.1	0.94			
Heterosexual females	15	2.5	1.0-6.5	0.06				5	0.4	0.1-0.9	0.03			
All others	4	6.9	2.0-24.0	<0.01				5	0.7	0.3-1.7	0.41			
Age at clinic entry (per 10 years)	36	2.4	1.9-3.1	<0.01	2.4	1.9-3.1	<0.01	50	2.3	1.8-2.8	<0.01	2.2	1.7-2.7	<0.01
Non-white race (ref = white)	36	0.7	0.3-1.4	0.26				50	0.6	0.4-1.1	0.13			
CD4+ lymphocyte count nadir (cells/μL)^§^														
≥200 (ref)	14	1.0			1.0			19	1.0			1.0		
<200	22	1.3	0.3-2.2	0.42	1.3	0.7-2.6	0.45	29	2.0	1.1-3.6	0.02	1.7	0.9-3.1	0.11
Missing	0	-	-	-	-		-	2	1.4	0.3-5.9	0.67	1.06	0.2-4.6	0.94
CD4+ lymphocyte count (cells/μL)^§^														
≥500 (ref)	12	1.0						11	1.0					
350-499	10	1.5	0.7-3.6	0.33				3	0.5	0.1-1.9	0.33			
<350	11	1.3	0.6-2.9	0.56				19	1.9	0.9-3.9	0.10			
Missing	3	0.4	0.1-1.6	0.20				17	0.8	0.4-1.7	0.57			
Log_10_ HIV-1 RNA level (per unit)^§^	27	0.6	0.4-1.1	0.08				33	1.0	0.7-1.3	0.83			
Cumulative time of log_10_ HIV RNA ≥ 5.0 (per 6 months)^§^	36	0.8	0.6-1.2	0.31				50	0.9	0.7-1.2	0.52			
Year of clinic enrollment	36	1.0	0.9-1.1	0.84	1.0	0.9-1.1	0.96	50	1.0	0.9-1.1	1.0	1.0	0.9-1.1	0.77
Hepatitis C virus infection	34	2.0	0.9-4.7	0.10				50	1.3	0.7-2.7	0.41			
Hepatitis B virus infection	32	0.5	0.1-3.5	0.47				50	1.2	0.4-3.4	0.68			
History of tobacco use	16	1.1	0.4-3.0	0.83				29	1.6	0.7-3.8	0.25			
Cancer prior to clinic entry	36	3.7	0.9-15.6	0.07	3.1	0.8-12.8	0.12	50	4.3	1.7-10.9	<0.01	2.2	0.8-6.1	0.14
ART exposure prior to clinic entry	36	1.5	0.8-3.0	0.23				50	1.6	0.9-2.8	0.09			

^§^Time-updated covariate.

^#^Number of events included in model.

Abbreviations:

INI: Instituto Nacional de Infectologia Evandro Chagas, Fundação Oswaldo Cruz, Rio de Janeiro, RJ, Brazil.

VCCC: Vanderbilt Comprehensive Care Clinic, Nashville, TN, USA.

HR: hazard ratio.

aHR: adjusted hazard ratio.

CI: confidence interval.

MSM: men who have sex with men.

IDU: injection drug use.

ART: antiretroviral therapy.

Lastly, all analyses were repeated using different windows of prevalent diagnoses in the sensitivity analyses presented in Additional file 3: Table S3. The differing prevalence windows had the greatest effect on number of incident ADCs. However, whether cancer diagnoses made after day zero, ten, or 30 following clinic entry were included, results for the analyses were similar.

## Discussion

This study is the first to describe the cancer epidemiology in a Brazilian HIV-infected cohort with comparisons to that of the Brazilian general population and a US HIV-infected cohort. Overall rates of ADCs appeared to be decreasing and rates of NADCs remained constant, a trend similar to that seen in the US cohort. Additionally, risk of specific types of cancer and predictors of cancer were similar between the two cohorts.

The decreasing rates of ADCs in both cohorts appeared to be driven by declining rates of KS. In the US, rates of KS were noted to decrease even prior to the availability of highly active ART [2, 20]. In Brazil, some studies have also suggested a decrease in the incidence of KS since 1996 [21]. In our study, incidence of KS was increased in the INI cohort compared to the VCCC cohort. This difference may have been a result of delayed entry into care or decreased access to ART, evidenced by the lower median CD4+ lymphocyte count at clinic entry and lower rate of ART receipt for INI. Studies examining human herpesvirus-8 prevalence in Brazil and elsewhere in Latin America have concluded a background infection risk similar to that of the U.S (0–6.6% of blood bank donors); however prevalence of human herpesvirus-8 in HIV-infected populations in Brazil has not been reported [22]. That the SIR for KS was lower for INI compared to VCCC in this study is likely a reflection of methodological differences in their calculation (the VCCC SIR was calculated using KS rates prior to the HIV epidemic and the INI SIR was calculated using contemporary KS rates, likely influenced by a majority of AIDS-associated cases of KS) [23]. At both sites where active cervical cancer screening programs are present, rates of cervical cancer were low and risk was not above expected for the respective general populations. Unlike KS and NHL, risk of cervical cancer among HIV-infected women has been most consistently reduced – and normalized to that of the general population – with cervical dysplasia screening and treatment programs rather than access to ART [24, 25].

While rates of ADCs appeared to be decreasing at both sites, rates of NADCs were constant. The increasing proportion of non-infectious morbidity and mortality in HIV-infected populations has been observed in both the US and Brazil [1, 15–20, 26]. In high-income countries, a number of malignancies have been observed to occur in HIV-infected populations at high rates, including anal cancer, lung cancer, Hodgkin lymphoma, liver cancer, and non-melanoma skin cancers [2]. Unlike KS and NHL, risks of these cancers have not consistently been associated with degree of immunosuppression [5, 27]. Among the NADCs diagnosed in the INI, risk of anal cancer was more than 60-fold greater than in the general population, likely related to men who have sex with men and high rates of human papillomavirus infection. In 2010, INI began screening for anal dysplasia; however, only two of the seven cases of anal cancer in this study were diagnosed that year. In 2014, anal dysplasia screening was also implemented at VCCC. These screening programs aim to reduce the excess anal cancer observed at both locations. Finally, rates of laryngeal cancer and lung cancer were also increased in the INI cohort, though not quite meeting statistical significance, and were likely related to the high tobacco use reported in the cohort. These cancers have also been observed to occur at increased rates in HIV-infected populations in the US [5, 28, 29]. Smoking cessation programs for harm reduction due to tobacco-related cancers is needed in both countries as the HIV-infected populations continue to age.

Examination of predictors of ADCs and NADCs yielded remarkably similar results for both cohorts. For both cohorts, female sex was associated with decreased risk of ADCs. This effect was likely due to increased risk of human herpesvirus-8 infection and KS among men who have sex with men, a pattern observed in previous studies from the U.S. and Brazil [21, 30]. Among the HIV disease markers that we examined, time-varying CD4+ lymphocyte count remained statistically associated with risk of ADC in multivariable models for both sites. In the multivariable model for INI, increased age at clinic entry was also associated with a small but significant increased risk of ADC. Previous studies have demonstrated an association between human herpesvirus-8 prevalence and age, which could explain this finding [31]. Further study of human herpesvirus-8 epidemiology among HIV-infected individuals in Brazil is needed to explore this relationship.

In examination of NADCs, age was the only significant predictor of risk in multivariable analyses for both cohorts. Unlike the strong association for risk of KS and NHL, CD4+ lymphocyte count has not been consistently associated with risk of NADCs [4, 8, 32–34]. In our study, risk of NADC was not associated with time-varying CD4+ lymphocyte count in univariable analysis at either site. Among patients at VCCC, low CD4+ lymphocyte nadir (<200 cells/μL) was associated with a two-fold increased risk of NADCs in univariable analysis but was not statistically significant in multivariable analyses adjusting for age.

Our study has a number of limitations. We aimed to describe incidence of cancers at both sites and sought to avoid misclassification of prevalent cases by excluding diagnoses occurring the first ten days of clinic follow-up. It is possible that some of the cancers included in this study reflect prevalent rather than incident cases; however, our analyses using a longer window (30 days) yielded similar results. Our study of incidence trends also failed to show a statistically significant decline in ADCs during the early years (1998–2004) of the study period. This may have been due to the smaller amount of person-time contributed during that time or delayed uptake of ART. Another important limitation occurred in the calculation of SIRs for INI. Limited by data available through the regional cancer registries, we could not perform year-adjusted analyses as part of the SIR calculation, having incidence data available only for 2000–2003. However, while these limitations are acknowledged, these analyses reflect the first assessment of cancer risk among a HIV-infected cohort compared to risk in the Brazilian general population. The similarity of the results with those of the US cohort suggests the conclusions drawn from the SIRs are reasonable and likely reflect valid epidemiologic patterns in Brazil. Lastly, due to the small number of events in both cohorts, cancers were assessed in the Cox models as ADCs and NADCs, rather than as individual cancer types. By doing so, cancers without a known association with HIV or other infections (such as breast and prostate cancer) may have limited our ability to detect risk differences conferred by immunodeficiency, HIV-1 RNA, or co-infection by hepatitis viruses. Grouping NADCs into subgroups, including “infectious” and “non-infectious,” may still result in false epidemiologic conclusions and was, therefore, not performed [35]. Even with treating the cancers in groupings of ADCs and NADCs, the small number of events limited our power to detect statistical differences in important predictors (such as immunodeficiency). Our ability to examine predictors of cancers was also limited by the partial availability of tobacco history for both cohorts, an important risk factor for NADCs. Given the stronger association of the effects of ART (immune status and HIV-1 RNA) rather than ART itself, our Cox models did not include time-varying ART as a predictor of cancer. Thus, we cannot assess if differences in ART regimens either within or between cohorts affected risk of ADCs or NADCs.

In this comparative study of Brazilian and U.S. cohorts, we found similar trends in cancer incidence, risk compared to respective general populations, and predictors of malignancy between the two cohorts. As an upper-middle-income country, Brazil is unique in its history of early availability of ART. Recent studies of causes of death among HIV-infected persons in Brazil also have indicated an increasing proportion of non-infectious mortality, similar to that of high-income countries. Further study is needed to examine the ongoing excess risk of KS in Brazil despite available ART. Development of screening and prevention programs for malignancy is needed in both countries.

## Conclusions

This study is the first to examine thoroughly epidemiologic trends of AIDS-defining cancers and non-AIDS-defining cancers in an HIV-infected population in Brazil, with a referent US HIV-infected population for comparison. Similar to trends observed in the US setting, the cancer burden in the HIV-infected population in Brazil has shifted from ADCs to increasingly NADCs. A number of NADCs occurred at rates higher than expected in the general Brazilian population. Ongoing cancer screening and prevention, including early availability of ART, is need in both countries.

## Methods

This study included adult patients (age ≥ 18 years) of the HIV clinics of the Instituto Nacional de Infectologia Evandro Chagas (INI) of Fundação Oswaldo Cruz in Rio de Janeiro and the Vanderbilt Comprehensive Care Clinic (VCCC) in Nashville. Both single-site cohorts, these clinics provide comprehensive HIV-specialty and primary care to HIV-infected patients. At both clinics, patients are seen routinely every 3 to 6 months for follow-up care. Data for this study were abstracted and validated from medical records at each site. The study was approved by the Comitê de Ética em Pesquisa of INI and by the Vanderbilt University Institutional Review Board.

This retrospective analysis included only new patients who enrolled in care between January 1, 1998, and December 31, 2010. For inclusion, patients must have completed a minimum of 60 days of follow-up (INI) or two clinic visits within the first year of clinic entry (VCCC). Patient follow-up time was censored at death, December 31, 2010, or at the last clinic visit when death occurred more than one year after the last clinic visit or if the last clinic visit was more than one year before the end of the study period (December 31, 2010). Demographic and clinical characteristics of the cohorts were compared using Chi square and Wilcoxon rank sum tests.

The primary outcomes of interest were incident ADCs and NADCs. ADCs included Kaposi sarcoma (KS), non-Hodgkin lymphoma (NHL), and cervical cancer. NADCs included all other cancers. To exclude prevalent cases, cancers diagnosed during the first ten days of clinic follow-up were excluded. Sensitivity analyses with only the exclusion of cancers diagnosed the day of clinic entry and with the exclusion of cancers diagnosed during the first 30 days of follow-up were performed.

Trends in cancer incidence were examined by Poisson regression. Incidence of ADCs and NADCs were calculated as the number of malignancies divided by the total person-time of follow-up of patients enrolled in care each calendar year. Trends were examined by incidence rate ratios (IRR) per year.

To compare rates of individual cancer types with those of the respective general populations, standardized incidence ratios (SIRs) were calculated by dividing the observed by the expected number of cases [36]. For INI, data from Instituto Nacional do Câncer and Instituto Brasileiro de Geografia e Estatística were used to calculate age- and sex-adjusted rates of each cancer type using the 2000–2003 Belo Horizonte registry, as data for Rio de Janeiro are not collected (available at http://www2.inca.gov.br and http://www.ibge.gov.br). Belo Horizonte was selected because, like Rio de Janeiro, it is a capital city of a southeastern state in Brazil. For VCCC, data from Surveillance Epidemiology and End Results (SEER) were used to calculate age-, sex-, race-, and year-adjusted rates of each cancer type using the Atlanta registry, as data for Nashville are not collected (available at http://seer.cancer.gov). Given the strong association of KS with HIV infection, expected KS cases were calculated using the age- and sex-adjusted rates from SEER for 1975–1979 [23]. Atlanta was selected because, like Nashville, it is a capital city of a southeastern state in the US. SEER does not collect data on squamous and basal cell skin cancers so SIRs for these cancers could not be calculated for the VCCC cohort.

Stratified by cohort, Cox proportional hazards models were used to examine predictors of first ADCs and NADCs, excluding squamous and basal cell skin cancers. Patient follow-up time was censored after first cancer event. Baseline demographic and clinical variables were included. Tobacco use data, dichotomized into ever or never users, were from cross-sectional surveys completed previously by a subset of patients at each clinic site. Time-updated CD4+ lymphocyte count (cells/μL), log_10_ HIV RNA (copies/mL), CD4+ lymphocyte nadir (cells/μL), and cumulative follow-up time with recorded log_10_ HIV RNA ≥ 5.0 were also included (described below). Multivariable models included a selection of variables based upon physiologic relevance and the number of events to avoid over-fitting.

Time-updated CD4+ lymphocyte count and log_10_ HIV RNA were created by dividing follow-up time into intervals based upon laboratory events. Laboratory values were carried forward until the next available laboratory results or censoring. When intervals were more than six months, time was divided such that time beyond six months after a laboratory event was associated with missing values until the next laboratory event or censoring occurred. Time-updated CD4+ lymphocyte count nadir was recorded as the lowest absolute CD4+ lymphocyte count available. Time-updated cumulative follow-up time with log_10_ HIV RNA ≥ 5.0 was recorded as the total amount of follow-up time with HIV RNA level ≥ 100,000 copies/mL. Patients with missing data were labeled as no documented time with log_10_ HIV RNA ≥ 5.0.

All reported *P* values are two-sided. All analyses were performed using Stata 12.1 (Stata Corporation, College Station, Texas, USA).

### Consent

Data for this study was obtained systematically from medical records of observational, clinic-based research cohorts. Patients at INI provide written informed consent for prior to inclusion in the observational cohort. Observational data from patients at VCCC are gathered retrospectively and, as written informed consent is not feasible for all patients, stored and analyzed removed of all personal identifiers to protect patient privacy. The study was approved by the Comitê de Ética em Pesquisa of INI and by the Vanderbilt University Institutional Review Board.

## Electronic supplementary material

Additional file 1: Table S1: Incidence of AIDS-defining cancers by year and site. (DOCX 90 KB)

Additional file 2: Table S2: Incidence of non-AIDS-defining cancers by year and site. (DOCX 91 KB)

Additional file 3: Table S3: Sensitivity analyses results of exclusion of cancer diagnoses at the day of clinic entry, within the first ten days of follow-up, and within the first 30 days of follow-up to remove prevalent cases from analyses. (DOCX 34 KB)

## Abbreviations

ADC: AIDS-defining cancer
AIDS: Acquired immunodeficiency syndrome
ART: Antiretroviral therapy
HIV: Human immunodeficiency virus
INI: Instituto Nacional de Infectologia Evandro Chagas, Rio de Janeiro, Brazil
IRR: Incidence rate ratio
KS: Kaposi sarcoma
NADC: Non-AIDS-defining cancer
NHL: Non-Hodgkin lymphoma
CI: Confidence interval
SEER: Surveillance Epidemiology and End Results
SIR: Standardized incidence ratio
VCCC: Vanderbilt Comprehensive Care Clinic, Nashville, USA.
