# Supplementary material for: HIV and cancer: a comparative retrospective study of Brazilian and U.S. clinical cohorts
Source: Infect Agent Cancer. 2015 Feb 2;10:4. doi: 10.1186/1750-9378-10-4 (PMC4327947; doi:10.1186/1750-9378-10-4)
Supplement: Supplementary file 1 — Additional file 1: Table S1: Incidence of AIDS-defining cancers by year and site. (DOCX 90 KB) [file 13027_2014_514_MOESM1_ESM.docx]

**Supplemental Table 1: Incidence of AIDS-defining cancers by year and site**

|  | INI | | | VCCC | | |
| --- | --- | --- | --- | --- | --- | --- |
|  | N | Person-years | Incidence [95% CI] | N | Person-years | Incidence [95% CI] |
| All years: |  |  |  |  |  |  |
| 1998-2010 | 57 | 12333.79 | 4.6 [3.5-6.0] | 51 | 15327.17 | 3.3 [2.5-4.4] |
| By year: |  |  |  |  |  |  |
| 1998 | 2 | 52.26 | 38.3 [4.6-138.2] | 0 | 139.17 | 0 [0.0-26.5] |
| 1999 | 1 | 157.14 | 6.4 [0.2-35.5] | 2 | 350.70 | 5.7 [0.7-20.6] |
| 2000 | 1 | 272.65 | 3.7 [0.1-20.4] | 3 | 564.81 | 5.3 [1.1-15.5] |
| 2001 | 2 | 414.80 | 4.8 [0.6-17.4] | 6 | 778.42 | 7.7 [2.8-16.8] |
| 2002 | 1 | 546.50 | 1.8 [0.0-10.2] | 3 | 922.23 | 3.3 [0.7-9.5] |
| 2003 | 4 | 636.83 | 6.3 [1.7-16.1] | 3 | 1102.27 | 2.7 [0.6-8.0] |
| 2004 | 3 | 767.85 | 3.9 [0.8-11.4] | 5 | 1327.84 | 3.8 [1.2-8.8] |
| 2005 | 10 | 915.93 | 10.9 [5.2-20.1] | 8 | 1455.25 | 5.5 [2.4-10.8] |
| 2006 | 6 | 1163.07 | 5.2 [1.9-11.2] | 5 | 1589.22 | 3.1 [1.0-7.3] |
| 2007 | 8 | 1463.86 | 5.5 [2.4-10.8] | 6 | 1740.09 | 3.4 [1.3-7.5] |
| 2008 | 8 | 1742.89 | 4.6 [2.0-9.0] | 6 | 1875.95 | 3.2 [1.2-7.0] |
| 2009 | 8 | 2017.27 | 2.0 [1.7-7.8] | 2 | 1970.32 | 1.0 [0.1-3.7] |
| 2010 | 3 | 2182.75 | 1.4 [0.3-4.0] | 2 | 1510.91 | 1.5 [0.2-4.8] |

Incidence per 1,000 person-years. N refers to the number of AIDS-defining cancers diagnosed. AIDS-defining cancers include incident Kaposi sarcoma, non-Hodgkin lymphoma, and cervical cancer

Abbreviations used:

INI: Instituto Nacional de Infectologia Evandro Chagas, Fundação Oswaldo Cruz, Rio de Janeiro, RJ, Brazil

VCCC: Vanderbilt Comprehensive Care Clinic, Nashville, TN, USA

CI: confidence interval
