# Supplementary material for: HIV and cancer: a comparative retrospective study of Brazilian and U.S. clinical cohorts
Source: Infect Agent Cancer. 2015 Feb 2;10:4. doi: 10.1186/1750-9378-10-4 (PMC4327947; doi:10.1186/1750-9378-10-4)
Supplement: Supplementary file 2 — Additional file 2: Table S2: Incidence of non-AIDS-defining cancers by year and site. (DOCX 91 KB) [file 13027_2014_514_MOESM2_ESM.docx]

**Supplemental Table 2: Incidence of non-AIDS-defining cancers by year and site**

|  | INI | | | VCCC | | |
| --- | --- | --- | --- | --- | --- | --- |
|  | N | Person-years | Incidence [95% CI] | N | Person-years | Incidence [95% CI] |
| All years: |  |  |  |  |  |  |
| 1998-2010 | 45 | 12333.79 | 3.6 [2.7-4.9] | 82 | 15327.17 | 5.3 [4.3-6.4] |
| By year: |  |  |  |  |  |  |
| 1998 | 0 | 52.26 | 0 [0.0-70.6] | 1 | 139.17 | 7.2 [0.2-40.0] |
| 1999 | 1 | 157.14 | 6.4 [0.2-35.5] | 0 | 350.70 | 0 [0.0-10.5] |
| 2000 | 0 | 272.65 | 0 [0.0-13.5] | 6 | 564.81 | 10.6 [3.9-23.1] |
| 2001 | 2 | 414.80 | 4.8 [0.6-17.4] | 3 | 778.42 | 3.9 [0.8-11.3] |
| 2002 | 0 | 546.50 | 0 [0.0-6.8] | 7 | 922.23 | 7.6 [3.1-15.6] |
| 2003 | 5 | 636.83 | 7.9 [2.5-18.3] | 7 | 1102.27 | 6.4 [2.6-13.1] |
| 2004 | 1 | 767.85 | 1.3 [0.0-7.3] | 6 | 1327.84 | 4.5 [1.7-9.8] |
| 2005 | 6 | 915.93 | 6.6 [2.4-14.3] | 8 | 1455.25 | 5.5 [2.4-10.8] |
| 2006 | 7 | 1163.07 | 6.0 [2.4-12.4] | 5 | 1589.22 | 3.1 [1.0-7.3] |
| 2007 | 5 | 1463.86 | 3.4 [1.1-8.0] | 10 | 1740.09 | 5.7 [2.8-10.6] |
| 2008 | 3 | 1742.89 | 1.7 [0.4-5.0] | 6 | 1875.95 | 3.2 [1.2-7.0] |
| 2009 | 4 | 2017.27 | 2.0 [0.5-5.1] | 14 | 1970.32 | 7.1 [3.9-11.9] |
| 2010 | 11 | 2182.75 | 5.0 [2.5-9.0] | 9 | 1510.91 | 6.0 [2.7-11.3] |

Incidence per 1,000 person-years. . N refers to the number of non-AIDS-defining cancers diagnosed. Non-AIDS-defining cancers include non-melanoma skin cancers.

Abbreviations used:

INI: Instituto Nacional de Infectologia Evandro Chagas, Fundação Oswaldo Cruz, Rio de Janeiro, RJ, Brazil

VCCC: Vanderbilt Comprehensive Care Clinic, Nashville, TN, USA

CI: confidence interval
