# Supplementary material for: HIV and cancer: a comparative retrospective study of Brazilian and U.S. clinical cohorts
Source: Infect Agent Cancer. 2015 Feb 2;10:4. doi: 10.1186/1750-9378-10-4 (PMC4327947; doi:10.1186/1750-9378-10-4)
Supplement: Supplementary file 3 — Additional file 3: Table S3: Sensitivity analyses results of exclusion of cancer diagnoses at the day of clinic entry, within the first ten days of follow-up, and within the first 30 days of follow-up to remove prevalent cases from analyses. (DOCX 34 KB) [file 13027_2014_514_MOESM3_ESM.docx]

**Supplemental Table 3: Sensitivity analyses results of exclusion of cancer diagnoses at the day of clinic entry, within the first ten days of follow-up, and within the first 30 days of follow-up to remove prevalent cases from analyses.**

|  | INI | | | VCCC | | |
| --- | --- | --- | --- | --- | --- | --- |
|  | Day 0 | Day 10 | Day 30 | Day 0 | Day 10 | Day 30 |
| ADCs (all):  KS  NHL  NADCs (all):  Excluding squamous and basal cell skin cancers | 62  42  20  46  37 | 57  37  20  45  36 | 51  32  19  45  36 | 52  22  29  83  55 | 47  19  27  81  53 | 41  17  23  80  52 |
| Incidence Trends (IRRs) [95% CI]:  ADCs  NADCs | 0.92 [0.85-1.00]  1.00 [0.90-1.13] | 0.92 [0.84-1.00]  0.99 [0.89-1.12] | 0.90 [0.82-0.98]  1.00 [0.89-1.12] | 0.90 [0.85-0.96]  1.00 [0.93-1.07] | 0.90 [0.85-0.96]  1.00 [0.93-1.07] | 0.91 [0.83-1.00]  1.02 [0.96-1.08] |
| SIRs [95% CI]:  All ADCs  All NADCs | 26.8 [20.5-34.3]  1.5 [1.1-2.0] | 24.6 [18.7-31.9]  1.4 [1.1-1.9] | 22.0 [16.4-29.0]  1.4 [1.1-1.9] | 18.8 [14.1-24.7]  1.3 [1.0-1.7] | 17.0 [12.5-22.6]  1.3 [1.0-1.7] | 14.8 [10.6-20.1]  1.3 [0.9-1.7] |
| Multivariable Cox Model for ADCs (aHR) (*p* value):  Female sex (ref=male)  Age at clinic entry (per 10 years)  CD4 lymphocyte count (cells/μL)  > 200 (ref)  50-199  <50  Missing  Cumulative time of log_10_ HIV RNA > 5.0 (per 6 months)  Year of clinic enrollment | 0.3 (<0.01)  1.3 (0.01)  1.0  5.0 (<0.01)  11.1 (<0.01)  3.4 (<0.01)  1.2 (0.20)  1.0 (0.56) | 0.3 (<0.01)  1.3 (0.04)  1.0  5.8 (<0.01)  13.7 (<0.01)  3.9 (<0.01)  1.2 (0.31)  1.0 (0.67) | 0.2 (<0.01)  1.3 (0.04)  1.0  5.7 (*p* <0.01)  14.0 (*p* <0.01)  4.1 (0.01)  1.2 (0.32)  1.0 (0.85) | 0.3 (0.01)  0.8 (0.14)  1.0  2.6 (0.04)  9.7 (<0.01)  1.4 (0.42)  1.1 (0.50)  1.0 (0.54) | 0.3 (0.02)  0.8 (0.16)  1.0  2.9 (0.03)  9.5 (<0.01)  1.4 (0.39)  1.1 (0.55)  1.0 (0.56) | 0.3 (0.04)  0.8 (0.15)  1.0  3.5 (0.02)  12.3 (<0.01)  1.8 (0.21)  1.0 (0.71)  1.0 (0.34) |
| Multivariable Cox Model for NADCs (aHR) (*p* value):  Female sex (ref=male)  Age at clinic entry (per 10 years)  CD4 lymphocyte nadir (cells/μL)  > 200 (ref)  <200  Missing  Year of clinic enrollment  Cancer prior to clinic entry | 1.3 (0.38)  2.4 (<0.01)  1.0  1.2 (0.56)  -  1.0 (0.85)  3.0 (0.13) | 1.4 (0.31)  2.4 (<0.01)  1.0  1.3 (0.45)  -  1.0 (0.96)  3.1 (0.12) | 1.4 (0.32)  2.4 (<0.01)  1.0  1.3 (0.45)  -  1.0 (0.96)  3.0 (0.13) | 0.7 (0.36)  2.1 (<0.01)  1.0  1.7 (0.08)  1.1 (0.93)  1.0 (0.64)  2.3 (0.12) | 0.7 (0.38)  2.2 (<0.01)  1.0  1.7 (0.11)  1.06 (0.94)  1.0 (0.77)  2.2 (0.14) | 0.7 (0.46)  2.2 (<0.01)  1.0  1.7 (0.17)  1.1 (0.88)  1.0 (0.97)  2.1 (0.17) |

Abbreviations:

INI: Instituto Nacional de Infectologia Evandro Chagas, Fundação Oswaldo Cruz, Rio de Janeiro, RJ, Brazil

VCCC: Vanderbilt Comprehensive Care Clinic, Nashville, TN, USA

ADC: AIDS-defining cancer

NADC: non-AIDS-defining cancer

KS: Kaposi’s sarcoma

NHL: Non-Hodgkin lymphoma

IRR: Incidence rate ratio per year increase from 1998-2010

SIR: Standardized incidence ratio

CI: confidence interval

aHR: adjusted hazard ratio
